# Calstabin 2: An important regulator for learning and memory in mice

**DOI:** 10.1038/srep21087

**Published:** 2016-02-18

**Authors:** Qi Yuan, Ke-Yu Deng, Le Sun, Shaopeng Chi, Zhiguang Yang, Jun Wang, Hong-Bo Xin, Xiaoqun Wang, Guangju Ji

**Affiliations:** 1 National Laboratory of Biomacromolecules, Institute of Biophysics, Chinese Academy of Sciences, Beijing 100101, China; 2 Department of Physiology and Cellular Biophysics, Columbia University, New York, New York 10032, USA; 3 Institute of Translational Medicine, Nanchang University, Nanchang 330000, China; 4 State Key Laboratory of Brian and Cognitive Sciences, Institute of Biophysics, Chinese Academy of Sciences, Beijing 100101, China; 5 School of Pharmaceutical Sciences, Tsinghua University, Beijing 100084, China

## Abstract

Calstabin2, also named FK506 binding protein 12.6 (FKBP12.6), is a subunit of ryanodine receptor subtype 2 (RyR2) macromolecular complex, which is an intracellular calcium channel and abundant in the brain. Previous studies identified a role of leaky neuronal RyR2 in posttraumatic stress disorder (PTSD). However, the functional role of Calstabin2 in the cognitive function remains unclear. Herein, we used a mouse model of genetic deletion of Calstabin2 to investigate the function of Calstabin2 in cognitive dysfunction. We found that Calstabin2 knockout (KO) mice showed significantly reduced performance in Morris Water Maze (MWM), long-term memory (LTM) contextual fear testing, and rotarod test when compared to wild type (WT) littermates. Indeed, genetic deletion of Calstabin2 reduced long-term potentiation (LTP) at the hippocampal CA3-CA1 connection, increased membrane excitability, and induced RyR2 leak. Finally, we demonstrated that the increase in cytoplasmic calcium activated Ca^2+^ dependent potassium currents and led to neuronal apoptosis in KO hippocampal neurons. Thus, these results suggest that neuronal RyR2 Ca^2+^ leak due to Calstabin2 deletion contributes to learning deficiency and memory impairment.

Ca^2+^ is a ubiquitous intracellular messenger that controls multiple functions in both excitable and non-excitable cell types. Intracellular Ca^2+^ release occurs via ryanodine receptors (RyRs) and inositol (1,4,5)-trisposphate receptors (IP3Rs) on the sarcoplasmic reticulum (SR) or endoplasmic reticulum (ER). All three RyR isoforms are expressed in the brain, with RyR1 and RyR2 being the predominant isoforms in the hippocampus. Intracellular Ca^2+^ plays a crucial role in neuronal survival, regulating excitability, secretion, and memory^1^. Several studies have demonstrated that neuronal Ca^2+^ homeostasis is disturbed in Alzheimer’s disease (AD)^2^ and other diseases of cognitive dysfunction^3^. Accumulating evidence indicates that disturbances of neuronal Ca^2+^ homeostasis represent a key pathogenic mechanism underlying neuronal dysfunction in the brain^1^, play a role in neuronal degeneration and cell death^4^,^5^. A recent report indicated that neuronal RyR2 -mediated Ca^2+^ leak contributes to stress-induced cognitive dysfunction, which can be rescued by stabilizing RyR2-Calstabin2 interaction via genetic ablation of the RyR2 PKA phosphorylation site at serine 2808^6^, suggesting that Ca^2+^ homeostasis is important in maintaining neuron function.

Calstabin2, also known as FKBP12.6, is a critical regulatory subunit of the RyR2 macromolecular complex. Calstabin2 selectively binds to RyR2 and stabilizes the closed state of RyR2, preventing aberrant calcium leak and pathogenic dysfunction in muscle and neuronal function^7^,^8^,^9^,^10^. Numerous studies have demonstrated that PKA phosphorylation, oxidation or nitrosylation of RyR2 dissociates Calstabin2 from the channel, resulting in calcium leak^11^,^12^,^13^,^14^ followed by cardiac hypertrophy^8^, arrhythmia^15^, heart failure^16^, impairment of insulin secretion^17^ and stress-induced cognitive dysfunction^6^. In contrast, enhanced Calstabin2 binding to RyR2 can improve cardiac function^18^, prevent heart failure^19^ and attenuate arrhythmias^15^. However, the function of Calstabin2 in neurons remains unclear. Furthermore, Calstabin2 proteins are known to selectively associate with RyR2 channels^9^, and inactivation of the Calstabin2 gene in mice results in a characteristic alteration of the Ca^2+^ spark properties in cardiac and smooth myocytes^8^,^9^. Ca^2+^ sparks activate Ca^2+^ dependent potassium currents or spontaneous transient out currents (STOCs) in smooth myocytes^9^. The activation of potassium currents has been shown to result in neuron apoptosis and cell death^20^,^21^,^22^; in contrast, blocking Ca^2+^ activated potassium channels results in LTP increase and learning improvement^22^, implying that targeting Calstabin2 could have upstream therapeutic value within the cognition field. In addition, it has been reported that Calstabin1 (FKBP12), which shares 85% sequence identity with Calstabin2, regulates mTOR-Raptor interactions through binding of rapamycin and inhibiting the activity of the mammalian target of rapamycin (mTOR), a key regulator of aging^23^. Through this pathway, Calstabin2 can affect long-term potentiation (LTP), memory, and perseverative behaviors^10^. Taken together, these observations suggest a crucial role for Calstabin2 in the regulation of brain function, which has yet to be fully examined.

The ability to store long-term memories depends on a brain region called hippocampus, where RyR2 expression is enriched. Patients with damage to this region are unable to form new long term memories (LTM) of day-to-day events. Given its importance, numerous studies have focused on the hippocampus for understanding how its neuronal network supports memory. In the present study, we show that genetic deletion of murine Calstabin2 results in impaired learning and memory. Following RyR2-mediated calcium leak, intracellular Ca^2+^ homeostasis is disturbed and Ca^2+^ dependent potassium currents are activated, signaling downstream apoptotic pathways and resulting in hippocampal neuron death.

## Results

### Genetic deletion of Calstabin2 alters behavior conditioning in mice

**Figure 1:**
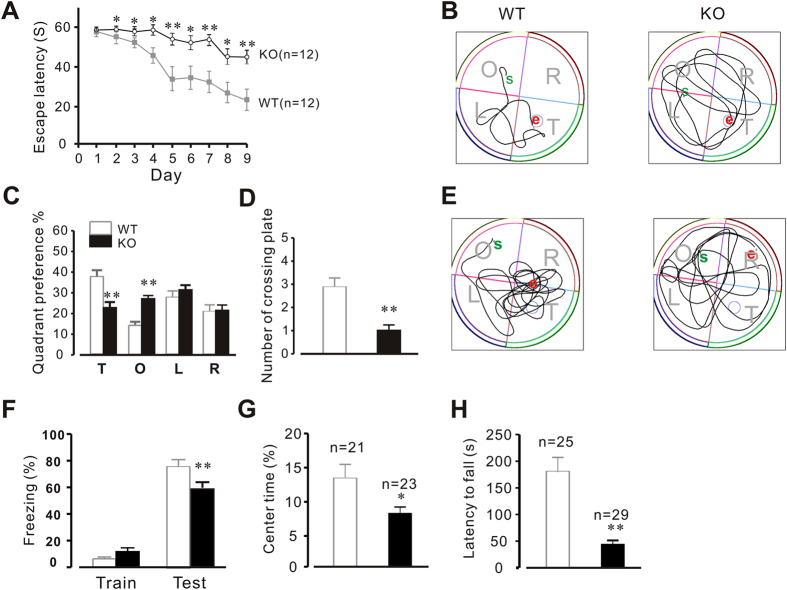
Deletion of Calstabin2 impairs learning and memory of mice. Morris Water Maze test was carried out in WT and Calstabin2 KO mice. (**A**) The average time needed to locate the platform during the training sessions. Since the 2nd day of training, Calstabin2 KO mice displayed a significant time delay in locating the platform compared to that of WT (n = 12 mice for each group). (**B**) Swimming paths of WT (left) and KO (right) mice during the probe trial session in the water Morris maze. (**C**) Quadrant preference of mice during the 1 min probe at day 10. Calstabin2 deletion significantly affected quadrant preference in KO mice. (**D**) KO mice displayed a reduced number of platform passing. (**E**) Map of mice crossing different quadrants after removing the platform. S and E indicate the start and end points, respectively. (**F**) Contextual fear conditioning test. Mean freezing behavior of WT and KO mice. F _(3,44)_ = 109.5, one way ANOVA. (**G**) Locomotor and exploratory activities measured in the open-field test. KO mice displayed more inactivity compared with WT mice. (**H**) Rotarod analysis on the third day of test. Data are shown as the means ± s.e.m. *p < 0.05 and **p < 0.01.

Our unpublished observations suggest that Calstabin2 knockout mice demonstrate oblivious behavior following fear stimulation, remaining still and unperturbed compared to WT littermates. To better understand the physiology underlying this unusual nonresponse, we conducted an MWM study designed to assess learning and memory function. As shown in Fig. 1, the average time needed to locate the platform was significantly delayed in Calstabin2 KO mice compared with that of WT littermates, beginning from the second day of the training session (Fig. 1A,B). Long-term training (post nine days) did not significantly improve the average platform location time in Calstabin2 KO mice (47.1 ± 4.31s), in comparison to WT mice (21.4 ± 4.39s). Additionally, quadrant preference analysis conducted at day 10 using a 1 min probe period demonstrated that deletion of Calstabin2 significantly affected the preference of mice for the target quadrant (Fig. 1C) and greatly reduced the number of platform passes (Fig. 1D) compared to controls. After the platform was removed from the pool, WT mice searched selectively around the training platform location, whereas Calstabin2 KO mice still searched equally across all four quadrants of the pool (Fig. 1E). Taken together, these data indicate a lack of both short term and long term memory retention.

Fear conditioning test is known to be associated with learning and memory^24^,^25^,^26^, thus, we examined the effect of Calstabin2 deletion on fear memory in WT and Calstabin2 KO mice. As indicated in Fig. 1F, both genotypes performed similarly during training. However, compared to the WT mice, Calstabin2-knockout mice displayed decreased freezing during LTM contextual fear memory testing (from 75.7 ± 3.42% (WT) to 58.2 ± 5.14% (KO), P < 0.01).

Next, we observed the effects of Calstabin2 deletion on animal activity. An open-field test was conducted to determine if locomotor and exploratory activities were perturbed in Calstabin2 KO mice. Mice were placed in open field and the time spent in the center of the entire open field was recorded. The results indicated that deletion of Calstabin2 reduced the activity of mice compared with WT controls (from 13.7 ± 2.02 (WT) to 8.26 ± 0.98 (KO), P < 0.05; Fig. 1G). To evaluate the effects of Calstabin2 deletion on motor coordination activity, an individual mouse was placed on a rotating drum. Measuring the time that each animal was able to maintain its balance walking on top of the rod, the rotarod test revealed that time lapse was significantly shortened in Calstabin2 KO mice compared to WT mice (from 178.42 ± 28.53 s to 43.49 ± 7.92 s, n = 25–29, P < 0.01, Fig. 1H). Together, these results suggest that genetic deletion of Calstabin2 leads to a remarkable decrease in memory retention and motor coordination.

### Deletion of Calstabin2 results in alteration of the electrophysiological properties of hippocampal neurons

**Figure 2:**
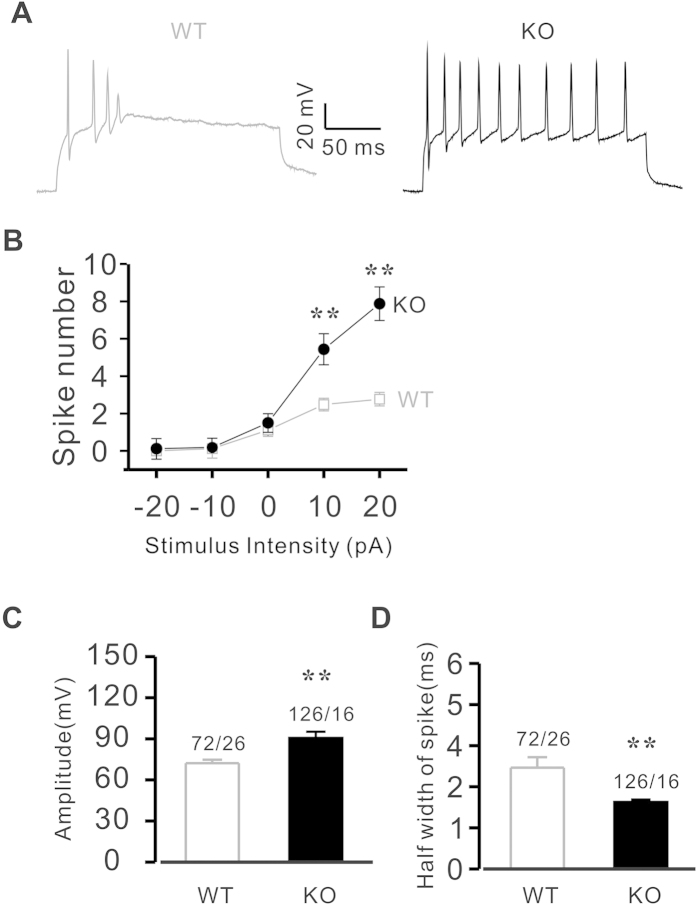
Alteration of excitability in Calstabin2 deficient hippocampal neurons. (**A**) Representative recordings of APs in single hippocampal neurons from WT (left) and KO (right) mice. Deletion of Calstabin2 increased the spike firing frequency (**B**) and the peak amplitude (**C**) of APs, but the duration of the AP was shortened (**D**). Data are shown as the means ± s.e.m. *p < 0.05 and **p < 0.01.

**Figure 3:**
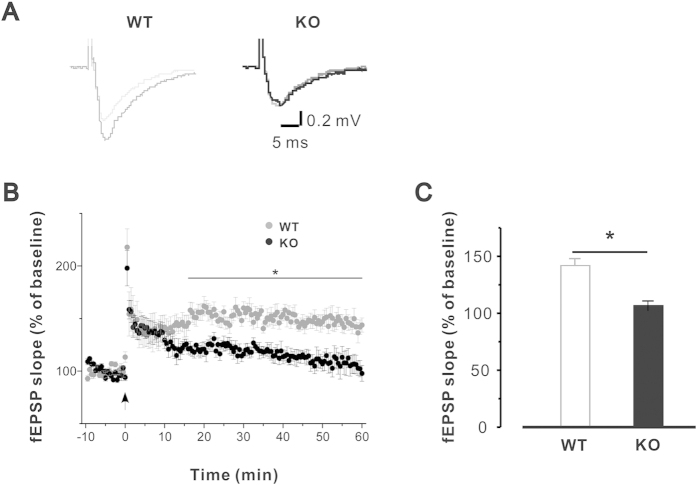
Genetic deletion of Calstabin2 reduces LTP in hippocampal neurons. (**A**) Representative experiments from WT (left) and KO mice (right) show averaged fEPSP comparing baseline and measurements taken in the last 5 min. (**B**) Time-course of fEPSP slope (mean ± s.e.m.) from WT (black) and KO mice (grey). The arrow indicates timing of burst stimulation. (**C**) Quantification of change in fEPSP slope following burst stimulation, showing the average responses to the last 5 minutes in control and mutant mice (mean ± s.e.m.). *p < 0.05 compared with controls.

Given that behavior is controlled by the hippocampal zone of the brain, one of the downstream consequences of increased RyR-mediated Ca^2+^ leak could be abnormal action potential conduction in hippocampal pyramidal neurons^27^. Mouse hippocampal neurons were isolated to examine the effect of Calstabin2 deletion on their excitability. As shown in Fig. 2, action potentials (APs) were significantly altered in Calstabin2 KO hippocampal neurons. The spike firing frequency for Calstabin2 KO mice was increased to 5.4 ± 0.8 times from 2.5 ± 0.3 times at 10 pA (P < 0.01) and to 7.9 ± 0.9 times from 2.8 ± 0.3 times (P < 0.01) at 20 pA, respectively (Fig. 2B). Further analysis of the results indicated that deletion of Calstabin2 caused an increase in the peak amplitude of Aps (Fig. 2C), but shortened the duration of spikes (Fig. 2D). To determine the role of Calstabin2 deletion on the alteration of learning and memory of mice, postsynaptic plasticity of hippocampal neurons was assessed by electrophysiological recordings of long-term potentiation (LTP) of hippocampus, which is required for learning and memory, in acute hippocampal slices prepared from both WT and Calstabin2 KO mice. As shown in Fig. 3, the slope of eovoked fEPSP in KO mice decays gradually and shows significant difference compared with WT mice from 26 min after stimulation. Figure 3C indicated, at the time of 60 min, fEPSP slope was significantly reduced in Calstabin2 KO mice (107.4 ± 8.96%, 6 slices from 5 mice) compared to WT mice (146.2 ± 5.24%, 5 slices from 5 mice, p < 0.05 by one-way ANOVA), confirming that Calstabin2 deletion results in murine cognitive dysfunction.

### Deletion of Calstabin2 alters Ca^2+^ signaling properties

**Figure 4:**
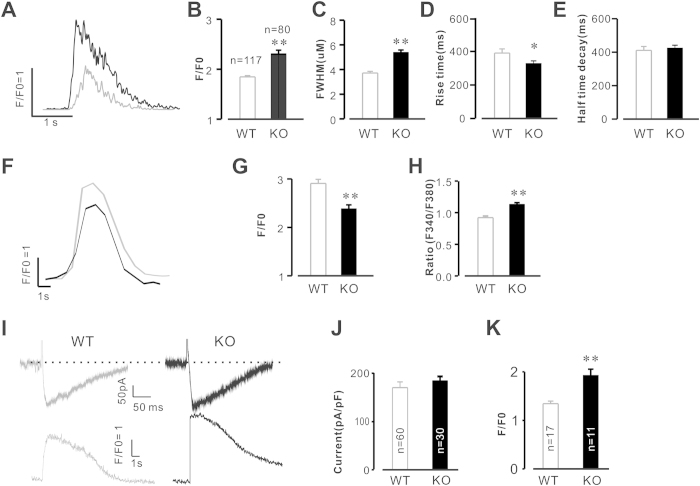
Genetic deletion of Calstabin2 alters Ca^2+^ release property in mouse hippocamtal neurons. (**A**) A typical recording of spontaneous Ca^2+^ release event in WT (gray) and KO (black) hippocampal neuron. Ca^2+^ release was significant altered by deletion of Calstabin2 (**B–E**). (**F**) Profiles of caffeine (10 mM) induced Ca^2+^ release in WT (gray, n = 22 from 6 mice) and KO (black, n = 18 from 6 mice) hippocampal neurons; the ER Ca^2+^ content was significantly decreased by deletion of Calstabin2 (**F**,**G**); intracellular resting Ca^2+^ was greatly increased in KO cells (**H**). (**I**) Deletion of Calstabin2 increases calcium induced calcium release (CICR) in mouse hippocampal neurons. The cell membranes were depolarized to 0 mV from the hold potential −50 mV with a depolarization duration of 250 ms. Calstabin2 KO did not affect the amplitudes of Ica (Ca, upper), while the peak of the intracellular Ca^2+^ releases was significant increased (lower). (**J**,**K**) Summary data of ICa and [Ca^2+^ ]i. Data are shown as the means ± s.e.m.; *P < 0.05 and **p < 0.01.

Previous studies indicated that RyR2 channel leakage results in perturbations of intracellular Ca^2+^ and impairs learning and memory^6^. Accordingly, we hypothesized that deletion of Calstabin2 disturbs intracellular Ca^2+^ stability and leads to impaired learning and memory. Therefore, we examined Ca^2+^ signaling dynamics in hippocampal neurons. As shown in Fig. 4A, the results of Ca^2+^ line-scan imaging demonstrated that deletion of Calstabin2 significantly altered the properties of Ca^2+^ release in hippocampal neurons. The peak Ca^2+^ was increased to 2.6 6 ± 0.21 from 1.71 ± 0.05 (Fig. 4A,B, P < 0.01); the full width at half maximum (FWHM) of Ca^2+^ releases was increased to 5.43 ± 0.26 μM from 3.71 ± 0.05 μM (Fig. 3C, P < 0.01); rise time was decreased to 326.6 ± 24.6ms from 390.2 ± 24.6ms (Fig. 4D, P < 0.05).

Next, we sought to determine if ER Ca^2+^ content is affected by Calstabin2 deletion in hippocampal neurons. After treatment with 10mM caffeine, ER Ca^2+^ content was significantly reduced in hippocampal neurons compared to that of WT cells (from 3.83 ± 0.22 to 2.71 ± 0.21, P < 0.01, Fig. 4F,G); correspondingly, the resting Ca^2+^ in the cytosol was remarkably increased in Calstabin2 null cells compared to that of WT (from 0.91 ± 0.03 to 1.12 ± 0.03, P < 0.01, Fig. 4H); this suggests that passive, RyR2-mediated Ca^2+^ leak occurred as a result of the Calstabin2 genetic deletion in hippocampal neurons. Furthermore, to simultaneously record the membrane Ca^2+^ currents and the intracellular Ca^2+^ release events, a combined patch-clamp and confocal microscopy technique was used. Our results indicated that L-type Ca^2+^ currents were similar in Calstabin2 KO and WT hippocampal neurons, but the peak of Ca^2+^ release from ER was significantly augmented in Calstabin2 null hippocampal neurons compared to that of WT (from 1.31 ± 0.05 to 1.96 ± 0.14, P < 0.01, Fig. 4K), suggesting that Calstabin2 KO RyR2 Ca^2+^ release channels were sensitized due to the genetic deletion. Taken together, these results indicate that deletion of Calstabin2 leads to RyR2 Ca^2+^ leak, which subsequently results in disturbances to hippocampal intracellular Ca^2+^ concentration.

### Genetic deletion of Calstabin2 activates Ca^2+^ dependent K^+^ currents in hippocampal neurons

**Figure 5:**
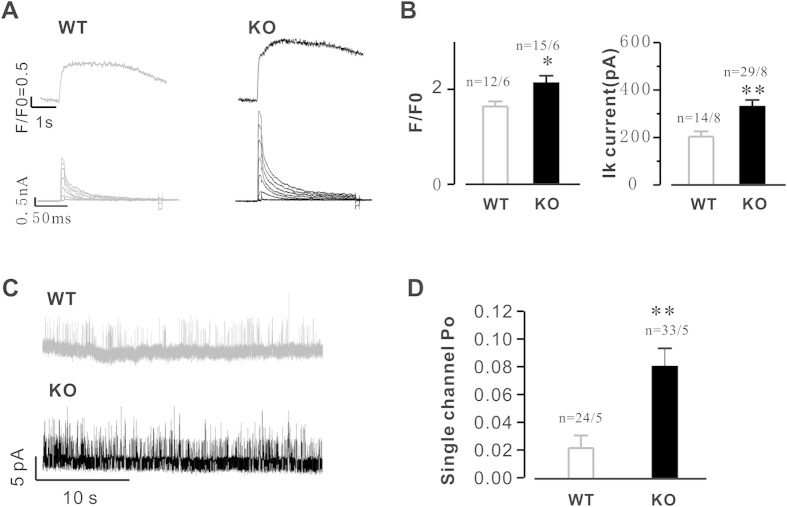
Genetic deletion of Calstabin2 activates Ca^2+^ dependent K^+^ currents in hippocampal neurons. (**A**) Simultaneous recording of Ca^2+^ release events and K^+^ currents using combined patch-clamp/confocal microscopy technique. Representative recordings of Ca^2+^ transients (upper) and K^+^ currents (IA, lower) in WT (left) and KO (right) hippocampal neurons. (**B**) Summary of the peak Ca^2+^ (left) and K^+^ currents (right). (**C**) Single channel recordings of spontaneous transient outward currents in WT and KO mouse hippocampal neurons. (**D**) Summary of data in C. Data are shown as the means ± s.e.m.; the numbers indicate cells/mice; *p < 0.05 and **p < 0.01.

K^+^ channels are essential for the function of the nervous system, where they modulate the shape and frequency of APs and consequently, neurotransmitter release. K^+^ channel inhibition has the potential to cause neuronal dysfunction by affecting the membrane potential of the cell, reducing repolarization and leaving the neuron susceptible to hyperexcitability. Excessive K^+^ efflux promotes apoptosis in hippocampal neurons^2^. To determine if hippocampal neuron apoptosis underlies the behavioural differences observed between Calstabin2 KO and WT mice, we examined Ca^2+^ -dependent or Ca^2+^ activated potassium currents in hippocampal neurons. The results from the combined patch-clamp and confocal microscopy study indicated that deletion of Calstabin2 increased peak Ca^2+^ to 2.14 ± 0.22 (KO) from 1.61 ± 0.11 (WT) (Fig. 5A, upper and B left, P < 0.05); correspondingly, the I_A_ currents were increased to 329.8 ± 28.2 pA/pF (KO) from 203.8 ± 21.9 pA/pF (WT) (Fig. 5A, lower, and B right, P < 0.01). Furthermore, single channel currents were also recorded with a holding potential at +10 mV, and the results demonstrated that the Calstabin2 deletion significantly increased the frequency of spontaneous transient outward currents (STOCS) in the Ca^2+^ activated potassium currents, from 0.02 ± 0.01s in KO versus 0.08 ± 0.01s in WT, P < 0.01 (Fig. 5C,D). These results indicate that deletion of Calstabin2 leads to alteration of the Ca^2+^ activated potassium currents in hippocampal neurons.

### Genetic deletion of Calstabin2 results in hippocampal neuron apoptosis

**Figure 6:**
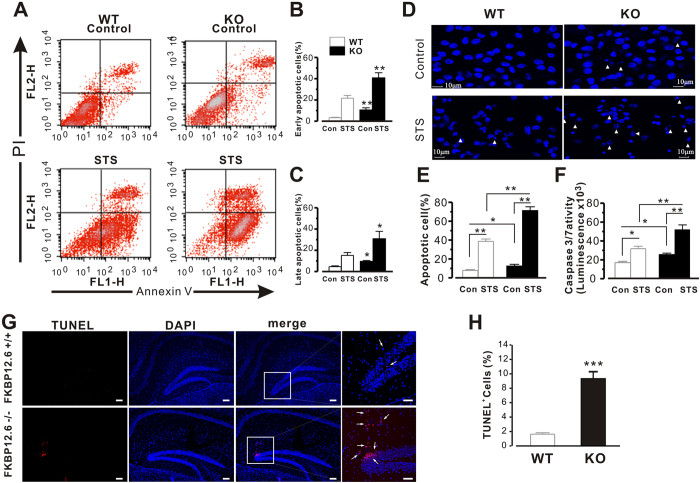
Calstabin2 deletion results in hippocampal neuron apoptosis. (**A**) Analysis of PI and annexin V binding of WT and KO hippocampal neurons 2 hours before and after treatment with staurosporine (1 μm). The plots indicate the proportion of live cells (annexin V-negative, PI- negative), early apoptotic cells (annexin V-positive, PI-negative) and dying cells (annexin V-positive, PI-positive); (**B**,**C**) are the summary data of early and late apoptotic cells in WT and Calstabin2 KO hippocampal neurons (F_(3,38)_ = 22.5 in (**B**) F_(3,38)_ = 6.8 in (**C**) one way ANOVA). (**D**) Representative images of STS-induced apoptosis in hippocampal neurons. Hippocampal neurons from WT and Calstabin2 KO mice were exposed to 1 μM STP for 2 hours and fixed with 4% paraformaldehyde, and their nuclei were stained by DAPI. Apoptosis was quantified as the percentage of cells with fragmented nuclei. White arrow heads indicate apoptotic and fragmented nuclei. Summary of the total cell death rate (**E**) and caspase-3/7 protease activity (**F**) induced by STS (F_(3,49)_ = 86.2; F value of STS- WT and KO = 15.36 in (**E**) F_(3,49)_ = 19.5 in (**F**) one way ANOVA). (**G**) TUNEL staining of brain sections indicated that Calstabin2 KO mice exhibited significantly higher rates of cell death compared to WT littermates. Data are presented as mean ± S.E. for four separate experiments. *p < 0.05, **p < 0.01 and ***P < 0.001 compared with control.

The significant neuronal loss in the hippocampus of the brain is believed to lead to clinical symptoms, such as cognitive decline. As cytoplasmic loss of K^+^ can enable protease and nuclease activation during apoptosis^28^ and deletion of Calstabin2 activate Ca^2+^ dependent potassium currents, we investigated whether apoptosis or cell death occurred due to the loss of hippocampal Calstabin2. To assess cell death, the cultured hippocampal neurons were exposed to staurosporine (STS, 1 μM) and double stained with Annexin V and propidium iodide (PI); neurons were analysed by a flow cytometry-based apoptosis assay. Figure 6A presents PI and Annexin V binding of WT and KO hippocampal neurons, 2 hours before and after treatment with STS. The plots show the proportion of live cells (annexin V-negative, PI- negative), early apoptotic cells (annexin V-positive, PI-negative) and late apoptosis cells (annexin V-positive, PI-positive). Early and late apoptosis were significantly increased in both baseline and STS treated hippocampal neurons derived from Calstabin2 KO mice compared with WT (Fig. 6B,C). The number of early apoptosis neurons was increased to 9.52 ± 2.21 (KO) from 3.31 ± 0.43 (WT) in control groups, and increased to 39.5 ± 4.91 (KO) from 21.7 ± 2.62 (WT) in STS treated groups respectively (Fig. 6B). The number of late apoptotic neurons was increased to 8.43 ± 1.16 (KO) from 4.61 ± 0.71 (WT) in control groups, and increased to 29.7 ± 7.11 (KO) from 15.2 ± 2.63 (WT) in STS treated groups (Fig. 6C). As seen in Fig. 6D, the total apoptosis rate of cells was increased to 12.4 ± 1.92 (KO) from 7.81 ± 0.98 (WT) in controls (P < 0.05), and to 54.3 ± 3.12 (KO) from 38.6 ± 2.51 (WT) in STS treated groups (P < 0.01). To confirm the rate of hippocampal neuron apoptosis, the activity of caspase 3 was analyzed. As shown in Fig. 6F, caspase 3/7 activity was greatly increased in Calstabin2 KO neurons compared to WT cells (from 17.1 ± 1.34 in WT to 25.5 ± 1.56 in KO, P < 0.05); the activity of caspase 3/7 in cultured hippocampal neurons increased to 51.7 ± 5.32 (KO) from 31.9 ± 2.23 (WT) after treatment with STS (P < 0.01). Furthermore, we also conducted these experiments on brain slices fixed to slides. As shown in Fig. 6G, TUNEL staining of brain sections indicated that Calstabin2 KO mice exhibited significantly higher rates of cell death compared to WT littermates.

**Figure 7:**
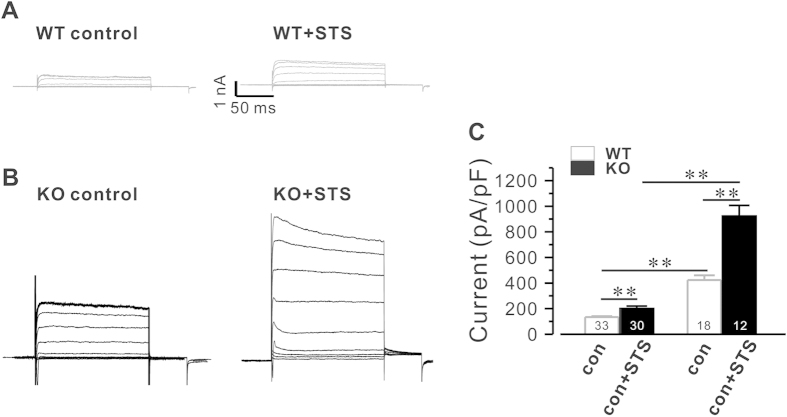
Deletion of Calstabin2 augments staurosporine induced K^+^ currents in hippocampal neurons. Outward K^+^ currents (Ik) were elicited by stepping to a conditioning voltage by depolarizing from −40 mV to +60 mV in increments of 10 mV. 0.5 nM STS was introduced through patch pipettes. (**A**) Sample of Ik recorded from WT hippocampal neurons without (left) or with (right) STS. (**B**) Representatives of Ik recorded from Calstabin2 KO cells in presence (right)) or absence (left) of STS. (**C**) Summary of Ik. Data are presented as mean ± S.E. for four separate experiments (F _(3,90)_ = 105.8. one way ANOVA) **p < 0.01 compared with control.

To further confirm that excessive K^+^ efflux promotes apoptosis in hippocampal neurons and STS induces significant apoptosis in Calstabin2 null hippocampal neurons, we examined the relationship between Ik, STS, Calstabin2 deletion and apoptosis of hippocampal neurons. Figure 7 shows that after treatment of cultured hippocampal neurons with STS (1 μM), I_k_ was remarkably increased in both WT and KO cells compared with controls (from 134. ± 6.99 pA/pF to 200.2 ± 19.4 pA/pF (WT, P < 0.01) and from 423.6 ± 36.9 pA/pF to 922.7 ± 83.5 pA/pF (KO, P < 0.01), respectively). Significantly, STS treatment induced much larger I_k_ in Calstabin2 KO hippocampal neurons than in WT cells (from 200.2 ± 19.4 pA/pF in WT to 922.7 ± 83.5 pA/pF in KO).

## Discussion

Intracellular calcium (Ca^2+^) homeostasis plays a crucial role in regulating neurological processes including synaptic transmission, secretion, excitability, learning, and memory^29^. Altered Ca^2+^ release from the ER is a common underlying mechanism of neurological disorders^2^,^30^,^31^,^32^,^33^,^34^. It has been reported that elevated intracellular Ca^2+^ is associated with cognitive dysfunction due to brain injury and neurodegeneration^35^,^36^. Elevated intracellular Ca^2+^ is associated with cognitive dysfunction via brain injury and/or neurodegeneration, as in the cases of Alzheimer’s disease and physiological aging^36^,^37^,^38^. Numerous studies have shown alterations of expression and function of RyRs in different murine models of alzhermer’s disease (AD), in human AD-affected brains, and in cells expressing familial AD-linked mutations on the β-amyloid precursor protein and presenilins^39^,^40^,^41^. Indeed, in the present study, we show that genetic deletion of Calstabin2, a protein that selectively binds and stabilizes the RyR2 Ca^2+^ release channel, caused cognitive dysfunction of mice (Fig. 1). Calstabin2 stabilizes the closed state of RyR2, preventing aberrant Ca^2+^ leak through the channel^35^. It is known that RyR2 (but not RyR1) is involved in stress-induced cognitive dysfunction, and that neuronal RyR2 remodeling by PKA hyperphosphorylation, oxidation, and nitrosylation plays an important role in cognitive dysfunction^6^.

The ability to store long-term memories is dependent upon the hippocampus of the brain, so patients with damage to this region are unable to form new LTM of day–to-day events^31^. Given their importance, the hippocampal neurons have been used to reveal the molecular mechanism underlying genetic deletion of Calstabin2 causing cognitive dysfunction of mice in the present study. Our electrophysiological study demonstrated that properties of the APs were significantly altered in the hippocampal neurons from Calstabin2 KO mice (Fig. 2), suggesting that abnormal action potential conduction is one of the downstream consequences of increased RyR2-mediated Ca^2+^ leak in hippocampal pyramidal neurons^24^. This result was further confirmed by our findings that deletion of Calstabin2 resulted in a significant alteration of Ca^2+^ release, wherein RyR2 channel leak led to sharp increases in the resting Ca^2+^ of hippocampal neurons (Fig. 4). Postsynaptic plasticity of hippocampal neurons reflects long-term hippocampal-type memory; CA3 and CA1 regions have revealed their distinct functions in learning and memory^42^,^43^, and the reduction of LTP at the hippocampal CA3-CA1 connection has been recognized as relating to deficits in learning and memory^44^. Indeed, in this study we demonstrated that genetic deletion of Calstabin2 resulted in a clear reduction of LTP (Fig. 3), further indicating that learning and memory were impaired in the null mice.

The significant synaptic and neuronal loss in the basal forebrain, hippocampus and cortex of the brain is believed to manifest as cognitive decline. Cell death or apoptosis is considered the main reason for neuronal loss in cognitive dysfunction^45^,^46^. Consistently, we show that genetic deletion of Calstabin2 results in significant hippocampal neuron apoptosis, both *in vitro* and *in vivo* (Fig. 6). Importantly, mitochondria play a central role in mediating apoptosis, generating reactive oxygen species (ROS), and maintaining intracellular calcium homeostasis; thus, mitochondrial abnormalities may be linked to the pathogenesis of neurological disorders^41^,^47^. Recently, calcium leak due to leaky RyR channels has been demonstrated to play a mechanistic role in determine mitochondrial dysfunction in muscle weakness^48^,^49^, heart failure^16^, atrial fibrillation^50^ and type 2 diabetes^17^. In the present study, we found leaky RyR2 channels and mitochondrial dysfunction might be a key link between Ca^2+^ disturbance and apoptosis. Besides, it has been reported that one of the major leading causes of neuronal death/apoptosis is the loss of intracellular K^+^ , due to the activation of Ca^2+^ activated potassium current^28^,^51^,^52^. In agreement with the reported results, we found that deletion of Calstabin2 led to increases in hippocampal potassium currents as a result of intracellular Ca^2+^ dysregulation (Figs 4 and 5). Excessive K^+^ efflux promotes apoptosis in hippocampal neurons, which was further confirmed by our findings that STS treatment induced much larger I_k_ in Calstabin2 KO hippocampal neurons than that in WT (Fig. 7).

One limitation of our study is that Calstabin2 knockout mice used are not hippocampal cell specific, but global knockout. Therefore, we cannot exclude the possibility that leaky RyR2 channels due to Calstabin2 deletion in other organs also contribute to the cognitive dysfunction observed in mice with Calstabin2 deletion. In addition, we did not perform the rescue experiments of overexpression of Calstabin2 in hippocampus in our KO mice by intrahippocampal injection of a lentiviral vector^53^. Such experiments could have helped distinguish between the roles of Calstabin2 in hippocampus function versus the other tissues. Furthermore, we plan to cross the Calstabin2 KO mice with mouse model of Alzheimer disease (APPPS1 mice) to further elucidate the function of Calstabin2 in neurological disease.

In summary, our study reveals that genetic deletion of Calstabin2 leads to leaky RyR2 channels. This results in perturbation of intracellular Ca^2+^ homeostasis in hippocampal neurons. The increases in intracellular resting Ca^2+^ activate Ca^2+^ dependent potassium currents, which causes hippocampal neuron apoptosis. This loss of hippocampal neurons led to significant cognitive dysfunction in Calstabin2 KO mice.

## Methods

### Animals

Calstabin2 KO mice were generated using homologous recombination to disrupt exon 3 of the Fkbp1b gene, as previously described^8^. All experiments were performed in accordance with the relevant guidelines and regulation that were approved by the Committee on Animal Care of Institute of Biophysics, Chinese Academy of Sciences, China. Mice were housed in sterile barrier facilities with equal day/night periods. Unless otherwise stated, paired littermates (both male and female) were used for the study to randomize genetic variation.

### Behavior studies

#### Morris water maze task

MWM was performed following the protocol described, with minor modifications (20). We used 8–12 week old WT and Calstabin2 KO mice in the present study. One day before the trial, the mice were released into a 122-mm diameter water tank and swum for 1 min. Learning trials were carried out for 9 consecutive days, with 2 trails per day. In every trail, mice were required to reach the platform in 1 min, and then were left on the platform for 15s. The value of each day was averaged from this 2 trails. A probe was conducted 24 h later after the mouse’s last trial. The platform was removed, and mice were allowed to return to the platform’s previous position for 1 min. Mice were tracked by a video camera (Sony) in both trials and probe. Collected data were analyzed by SMART 2.5 software (Panlab, Harvard Apparatus).

### Contextual fear conditioning

Training and testing were performed as previously published protocols with slight modifications^54^,^55^,^56^. In brief, mice were placed in a square chamber apparatus (L × W × H in cm, 25 × 25 × 40). The training sessions consisted of a 120s exploration period as baseline and followed by two times of CS-US (conditioned/unconditoned stimulus) pairings separated by one minute (tone 80 db white noise, 28 s duration; foot-shock intensity 0.2 mA, 2s duration). Testing sessions were performed 24 hours later. The mice trained before were placed in the same training chamber. After 2 min habituation, a sound was given for 3 min. Freezing behavior was defined as the absence of movement except for respiration (Fanselow, 1980) and was recorded by SMART v2.5 software (Panlab; Harvard Apparatus). Chambers were carefully cleaned using 70% ethanol between each trial.

### Open field test

Mice were individually placed in an open-field apparatus (L × W × H in cm, 90 × 90 × 45). No prehabituation were done in the arena. Mice were allowed to explore freely for 15 min and the movement was recorded by a video camera (Sony). Time spent in the center (seconds) and periphery (seconds) was recorded to test the locomotor activity level. Data were collected and analyzed using SMART v2.5 software (Panlab; Harvard Apparatus).

### Rotarod performance study

Motor coordination and balance were tested using an accelerating rotarod (YLS-4C, ShangHai Biowill Co.). The rotarod test was performed by placing mice on a rotating drum and measuring how long each animal was able to maintain its balance walking on top of the rod. The speed of the rotarod accelerated from 4 to 40 rpm over a five min period. Mice were subjected to three trials, with a maximum time of 5 min and a 15 min inter-trial test interval. 3 trials per animal were averaged.

### Field excitatory postsynaptic potentials

Rapid cervical dislocation followed by decapitation was carried out to sacrifice the mice. Transverse hippocampal slices (300  μm) were prepared using a vibratome (VT1200S, Leica) in oxygenated (95% O_2_ and %5 CO_2_) ice-cold sucrose-based artificial cerebrospinal fluid (sucrose-based ACSF) containing (in mM): 234 Sucrose, 2.5 KCl, 26 NaHCO_3_, 1.25 NaH2PO_4_, 11 D-Glucose, 0.5 CaCl_2_ and 10 MgSO_4_. The slices were kept in an incubating chamber filled with oxygenated ACSF (in mM: 126 NaCl, 3 KCl, 26 NaHCO_3_, 1.2 NaH2PO_4_, 10 D-Glucose, 2.4 CaCl_2_ and 1.3 MgCl_2_) at 34 °C. After a recovery period of at least 60 minutes, an individual slice was transferred to a recording chamber and was continuously superfused with oxygenated ACSF at a rate of 5 ml per minute at 30 ± 1 °C.

Field excitatory postsynaptic potentials (fEPSPs) were recorded using ACSF-filled glass pipettes (3–5 MΩ) placed at the stratum radiatum of area CA1. fEPSPs were evoked by using concentric bipolar electrode (WPI), which was used to stimulate the schaeffer collateral (SC) fibers with a brief current pulse (100 μs). The current pulse was delivered by stimulus isolation unit (ISO-Flex, A.M.P.I.) every 30s. The distance between stimulating electrode and recording pipette is 200–300 μm. An input-output curve was used to set the stimulating strength, which yielded 30–50% of the maximal slope. After obtaining baseline measurements for 30 minutes, LTP was induced using θ-burst stimulation (BS, burst containing 4 pulses at 100 Hz repeated at 5 Hz and two 10-burst trains separated by 20s). Evoked fEPSPs were recorded for 1 h after tetanization. Signals were filtered at 2 kHz and digitized at 100 kHz using Digidata 1440A (Molecular Devices). Data acquisition and slope measurement were carried out using pClamp 10.2 (Molecular Devices), pulse generation was achieved using Master 8 (A.M.P.I.).

### Neuronal cell culture

Hippocampal neurons were acutely dissociated and cultured according to a previous method^57^ with slight modification. Briefly, the hippocampal were dissected from neonatal mice (within 24 h after birth). The hippocampal neurons were dissociated by incubation (7 min, 37 °C and 5% CO_2_) in 0.25% Trypsin-EDTA (GIBCO) and triturated in DMEM (GIBCO) supplemented with 10% bovine serum (Hyclone). The resulting hippocampal cells were plated at a density of 2 × 10^5^ cells/cm^2^ onto polyL-lysine (Sigma, St. Louis, MO, USA) coated glass coverslips (Matsunami, Japan). The coverslips were then incubated at 37 °C in a humidified atmosphere of 95% O2 and 5% CO_2_. The media was replaced 7h later with Neurobasal TM—A Medium, B-27 (GIBCO) and 0.5 mM glutamine without antibiotic solution. After 48h culture the media was changed to Neurobasal and B-27. Hippocampal pyramidal neurons were identified morphologically by their large pyramidal shaped cell body with a thick stump of apical dendrite. Hippocampal neurons were cultured for 6 days *in vitro* (DIV). All experiments were performed at room temperature (22–25 °C).

### Patch Clamp Recordings

Whole cell currents and APs from hippocampal neurons were recorded with multi-clamp 200B, then inputted into pClamp 9 (Axon Instrument Inc., Foster CA, USA) for data acquisition and analyses. Voltage-clamp recordings were low-pass filtered at 2 kHz, and current-clamp recordings were low-pass filtered at 5 kHz. Pipettes were fire-polished to give a final resistance of 3–5 M Ω for whole-cell recording. Pipette and membrane capacitances were compensated for automatically with the amplifier. Series resistance compensation of 50–70% was employed routinely to reduce voltage error. Offset potentials were nullified directly before formation of the seal. Transients and leakage currents were recorded and digitally subtracted offline in all experiments using averaged recordings with hyperpolarizing impulses (100 ms voltage steps from −80 to −120 mV) that did not activate currents. Fresh pipette solution was filtered with 0.1 μM centrifuge filter. The pipette solution osmolarity was 295–305 mOs mol. The extracellular solution used was HANKS’ Balanced salts solution (HBSS, Sigma), containing (in mM) 1.3 CaCl_2_, 0.8 MgSO_4_, 5.4 KCl, 0.4 KH_2_ PO_4_, 136.9 NaCl, 0.3 Na_2_ PO_4_, 10 D-glucose and 4.2 NaHCO_3._ The intracellular solution for K^+^ currents and action potential recordings contains (in mM) 140 KCl, 2 MgCl_2_, 2 CaC_2_, 1 EGTA, 2 Na_2_ ATP and 10 HEPES at pH 7.3. For current clamp, neurons were held at potentials between −70 to −60 mV with steady injection of current. APs were evoked by injecting current of 200 ms duration, while amplitude was increased from −20 to 20 pA in steps of 10 pA. The components of voltage-dependent K^+^ currents were isolated using voltage-clamp protocols. The total K^+^ currents were recorded in response to depolarizing voltage steps from a holding potential of −90 mV to +70 mV in a step of 20 mV, which activated outward currents consisting of two major components: a fast transient I_*A*_ and a delayed rectifier I_k_ currents. When a 100 ms was prepulsed to −40 mV preceded the test pulses, almost all of the transient I_A_ current was inactivated, leaving the outward rectifier I_k_ current. The transient I_A_ currents were obtained by subtracting I_k_ from the total K^+^ currents.

To record Ca^2+^ channel currents, the chamber was perfused with a bathing solution containing (in mM): 132 Choline-Cl, 4 CaCl2, 5 Hepes, 1 MgCl_2_, 10 glucose, 4 CsCl, 20 TEA, 0.0005 TTX, pH adjusted to 7.4 with CsOH. The intracellular solution was (in mM):120 CsCl, 20 TEA, 5 EGTA, 5 MgATP, 5 Hepes, 1 EDTA, pH adjusted to 7.3with CsOH. The inward Na^+^ currents were blocked by TTX. In some cases, voltage-clamped cells were dialyzed with 10 μM Fluo-4 and simultaneously scanned to measure Ca^2+^ transients.

The signal calcium-activated potassium channel currents were recorded in cell attached mode, and cells were clamped at +30 mV. Currents were filtered at 500 Hz and digitized at 2 kHz. Pipette resistance was 5–7 MΩ. The intracellular solution was (in mM): 130 KCl, 2 MgCl_2_, 1 Na_2_ATP, 1.1 CaCl_2_, 1 EGTA, pH 7.3, and the extracellular solution was: 140 NaCl, 3 KCl, 2 CaCl_2_, 1 MgCl_2_, 10 HEPES, 0.0005 TTX, 1 4-aminopyridine (4-AP), 0.005 glybenclamide, and 10 glucose; pH adjusted to 7.4 with NaOH. TTX, 4-AP, and glybenclamide were included routinely in the K^+^ recording solution unless otherwise indicated. 4-AP (1 mM) was used to reduce voltage-gated K^+^ currents and unmask Ca^2+^ dependent K^+^ currents. A low concentration of 4-AP was chosen to minimize possible nonspecific blockade of other K^+^ currents, such as SK currents. Experiments were conducted at room temperature (22–25 °C). Extracellular recording solution contained a mixture of channel blockers: 4-AP, 1 mM, for transient voltage-gated K^+^ current; TTX, 0.0005 mM, for Na^+^ current; and glybenclamide, 0.005 mM, for ATP-sensitive K^+^ current. These agents were added routinely to the recording buffer unless otherwise stated.

### Measurement of Ca^2+^ Fluorescence

Hippocampal neurons were incubated with 10 μM Fluo-4 AM (Molecular Probes) for 10 min at room temperature and transferred into a recording chamber. Cells were then perfused with extracellular solution (see above) for 40 min. Fuo-4 fluorescence was recorded using Leica SP5 confocal microscope equipped with an argon laser (488 nm) at a magnification of 60x using an oil immersion objective (1.25 NA). For the measurements of SR Ca^2+^ load, 10 mM caffeine was added via pipette. Images were acquired either using x-t scan or x-y-t scan. Images were processed and analyzed using MATLAB 7.1 software (MathWorks) or ImageJ (Scioncorp).

### Resting Ca^2+^ measurement

Resting Ca^2+^ of hippocampal neurons were measured as previously described^7^. Briefly, cells were incubated with 10 μM Fura-2 AM (Invitrogen) for 45 min at 37 °C. After loading, the cells were washed several times and transferred to a recording chamber. Photometry measurements were made using a wide-field Olympus cell^R system, operated at an emission wavelength of 510 nm, with excitation wavelengths of 340 and 380 nm. The relative resting Ca^2+^ level (estimated by a ratio of 340 nm/380 nm) was recorded and data were analyzed using Olympus cell^R Software.

### Neuronal cell death assay

Postnatal Day 6 hippocampal neurons of both WT and KO mice were treated with 1 μM STS (Aldrich, St. Louis, Missouri) for 24 hours. To assay cell death, the cells in each group were fixed in 95% ethanol for 5 min, washed with phosphate-buffered saline, and stained with 4,6-diamino-phenylindole (DAPI, 5 μmol/L, Roche) for 15 min. Images of the cells were obtained by using Leica SP5 confocal. At least 200 nuclei were counted in 4 randomly chosen subfields within each neuronal culture to determine, based on the appearance of apoptotic bodies, the percentage of apoptotic cells.

### Flow cytometer analysis

Detection of apoptosis in cultured hippocampal neurons were performed according to the manufacturer’s instruction (Annexin V-FITC Apoptosis Detection Kit, Beyotime). Briefly, cells were washed for two times with PBS; 1–5 × 10^5^ cells were collected and suspended in 500 μl binding buffer followed by the addition of both Annexin V-FITC(5 μl) and PI(5 μl). After 5–15 min of dark incubation at room temperature, the cells were analysed using flow cytometer (Becton Dickinson) within 1h. After setting the gate around the hippocampal neuron population, the data were analyzed by CellQuest software (BD). The results were presented as two-colour fluorescent diagrams of cells stained with anti-annexin V-FITC and PI. Cell apoptotic rate was calculated as Annexin V-FITC positive staining for early apoptosis, and both Annexin V-FITC and PI positive staining for late apoptosis/death.

### Detection of caspase-3/7 protease activity

Cultured hippocampal neurons were cultured in 96-well plates and treated with 1 μM STS (Aldrich, St. Louis, Missouri). CaspaseGlo™ 3/7 Assay Kit (Promega) was applied to evaluate the activity of caspase-3/7 protease. After treatment with STS (24 hr), 100 μl of homogenous caspase-3/7 reagent was added to each well of the cultured neurons. The contents of wells were gently mixed and incubated at room temperature for 45 min. The luminescence intensity of each sample was measured with Veritas™ Microplate Luminometer (P/N 9100-000). Experiments were performed four times per each of the 12 mice.

### TUNEL analysis

Mouse brains were fixed in 10% phosphate-buffered formaldehyde for 24 hours, dehydrated with ethanol and histoclear, and embedded in paraffin. Formalin-fixed, paraffin-embedded brain sections were deparaffinized in xylene three times for 10 min, followed by rehydration in graded alcohol (100%, 95%, 85%, and 70%), and then washed three times with PBS for 5 min. Brains sections (5 μm) were processed for Hoechst/terminal deoxynucleotidyl transferase-mediated biotinylated UTP nick end labeling (TUNEL), double labeled using *In Situ* Cell Death Detection kit as per the manufacturer’s instructions (Roche Molecular Biochemicals, Mannheim, Germany). The number of TUNEL-positive cells and total cells in brains tissue sections were counted under Leica SP5 confocal microscope.

### Data Analysis

All data are the mean ± SEM for at least 3 experiments. Data were analyzed with Origin 7.0 (Origin Lab, Northampton, MA, USA). Data were analyzed statistically using the unpaired Student’s t test, 2-tailed (for 2 groups), and 1-way ANOVA with Tukey-Kramer post hoc correction (for groups of 3 or more) unless otherwise. The criterion for a significant difference was *P < 0.05 and **P < 0.01.

## Additional Information

**How to cite this article**: Yuan, Q. *et al.* Calstabin 2: An important regulator for learning and memory in mice. *Sci. Rep.* **6**, 21087; doi: 10.1038/srep21087 (2016).
